# Performance of Broilers Fed with Maize Colonized by Either Toxigenic or Atoxigenic Strains of *Aspergillus flavus* with and without an Aflatoxin-Sequestering Agent

**DOI:** 10.3390/toxins11100565

**Published:** 2019-09-26

**Authors:** M. O. Samuel Aikore, Alejandro Ortega-Beltran, Daisy Eruvbetine, Joseph Atehnkeng, Titilayo D. O. Falade, Peter J. Cotty, Ranajit Bandyopadhyay

**Affiliations:** 1 Pathology and Mycotoxin Unit, International Institute of Tropical Agriculture (IITA), Ibadan 200001, Nigeria; s.aikore@cgiar.org (M.O.S.A.); a.beltran@cgiar.org (A.O.-B.); j.atehnkeng@cgiar.org (J.A.); t.falade@cgiar.org (T.D.O.F.); 2 Department of Animal Nutrition, Federal University of Agriculture, Abeokuta, PMB 2240, Alabata Road, Ogun State 110124, Nigeria; dayeruv@gmail.com; 3 USDA-ARS, Tucson, AZ 85701, USA; cottypj@gmail.com

**Keywords:** biocontrol, aflatoxin, poultry, maize, toxin binders

## Abstract

In warm agricultural areas across the globe, maize, groundnut, and other crops become frequently contaminated with aflatoxins produced primarily by the fungus *Aspergillus flavus*. Crop contamination with those highly toxic and carcinogenic compounds impacts both human and animal health, as well as the income of farmers and trade. In Nigeria, poultry productivity is hindered by high prevalence of aflatoxins in feeds. A practical solution to decrease crop aflatoxin content is to use aflatoxin biocontrol products based on non-toxin-producing strains of *A. flavus*. The biocontrol product Aflasafe^®^ was registered in 2014 for use in maize and groundnut grown in Nigeria. Its use allows the production of aflatoxin-safe maize and groundnut. A portion of the maize treated with Aflasafe in Nigeria is being used to manufacture feeds used by the poultry industry, and productivity is improving. One of the conditions to register Aflasafe with the national regulator was to demonstrate both the safety of Aflasafe-treated maize to avian species and the impact of Aflasafe as a public good. Results presented here demonstrate that the use of maize colonized by an atoxigenic strain of Aflasafe resulted in superior (*p* < 0.05) broiler performance in all evaluated parameters in comparison to broilers fed with toxigenic maize. Use of an aflatoxin-sequestering agent (ASA) was not sufficient to counteract the harmful effects of aflatoxins. Both the safety and public good value of Aflasafe were demonstrated during our study. In Nigeria, the availability of aflatoxin-safe crops as a result of using Aflasafe allows poultry producers to improve their productivity, their income, and the health of consumers of poultry products.

## 1. Introduction

Aflatoxins are highly toxic and carcinogenic compounds produced by *Aspergillus flavus* and closely related fungi in several crops [1]. Maize (*Zea mays* L.) and groundnut (*Arachis hypogea* L.) are amongst the most susceptible crops. Crop contamination with these potent compounds negatively affects both human and animal health, the income of farmers, and trade opportunities [2,3,4]. In the case of animals, when feeds are prepared with aflatoxin-contaminated crops, their productivity decreases drastically [5,6]. Poultry species are particularly sensitive even at low aflatoxin concentrations, with negative effects ranging from poor and delayed growth, high feed conversion ratio (FCR), deficient nutrient usage, immunosuppression, liver diseases, poor egg-shell quality, and high mortality, among other constraints [7].

In order to satisfy the nutritional requirements of avian species, their feeds typically contain >50% maize or groundnut [8]. Across sub-Saharan Africa (SSA), maize and groundnut are frequently contaminated with aflatoxins [2,9,10], making procurement of aflatoxin-safe crops/feeds a major challenge for poultry industries. In Nigeria, where poultry economy is growing rapidly [11], the proportion of feeds containing unsafe aflatoxin concentrations is high [12,13]. Few poultry producers can import aflatoxin-safe crops to manufacture safe feeds. Poultry producers may choose to use soybean as a substitute for groundnut, although this results in an elevated cost and requires additional processing to remove antinutritional compounds found in soybean [14]. Other producers incorporate aflatoxin-sequestering agents (ASAs) into feeds to attempt to limit both the availability of the toxins and their effects [15].

ASAs can be largely subdivided into three types: mineral, organic, and biological. The subdivision within each type, their mode of action, and their efficacy were recently reviewed [16]. The efficiency of ASAs differs considerably depending mainly on the chemical structure of the adsorbent [17]. Some ASAs were reported to significantly reduce aflatoxin effects in poultry species [18,19]. However, a main limitation of some ASAs is that their use generally results in higher FCR, and poultry species need to receive higher feed rations [16]. This incurs substantial additional cost to producers. Furthermore, accumulation of antibiotics at dangerous concentrations was detected in tissues of birds that consumed feeds amended with an ASA [20]. Although several ASAs are available and commonly used across the globe, only a few are approved by regulatory authorities. In Europe, bentonite is the sole ASA recommended by the European Commission as a mycotoxin binder for use in poultry, ruminants, and pigs [16]. In the United States (US), eight ASAs are registered for use in the state of Texas [21].

Substantial efforts were dedicated to make ASAs available to livestock producers. However, the most effective way to reduce livestock exposure to unsafe aflatoxin levels is to prevent aflatoxin contamination of the crops that will be processed into feeds. Aflatoxin-safe crops can become available using a suite of technologies that prevent aflatoxin formation before, during, and after harvest [22,23]. Among these technologies, a simple, safe, and cost-effective one employs atoxigenic (i.e., non-toxin-producing) strains of *A. flavus* as biocontrol agents to displace aflatoxin-producing strains [24]. Atoxigenic *A. flavus* strains contain natural deletions, insertions, or defects in one or more of the genes responsible for aflatoxin formation and, therefore, cannot produce the toxins [25,26]. In 11 SSA nations, several biocontrol products have been developed under the trade name Aflasafe through intensive collaboration among the International Institute of Tropical Agriculture (IITA), United States Department of Agriculture – Agricultural Research Service (USDA-ARS), and local national institutions [27,28]. The Aflasafe product developed for Nigeria was the first aflatoxin biocontrol product developed for an African nation and is the third one worldwide after two products developed for use in commercial crops in the US [27,29,30,31]. Aflasafe products are composed of sterile sorghum grains coated with spores of four atoxigenic strains of *A. flavus* native to nations where the product is intended to be used. Sorghum grains serve as both the carrier and nutritious source for the biocontrol strains [27]. When applied in the field before crop flowering, the biocontrol strains reproduce on the sorghum grains before reaching to the target crop. Once associated with the crop, the atoxigenic strains displace most of the toxigenic fungi residing in the treated field, resulting in little to no aflatoxins in the treated crop [27,29,30,32].

Maize and groundnut from Aflasafe-treated fields typically contain 70–99% less aflatoxins than crops from untreated fields [33]. Thus, incorporating crops from treated fields into feeds may offer an alternate option to ASAs. On the other hand, there is a potential concern that free-ranging poultry and other birds may be harmed when they forage on the formulated product after it is broadcasted in the field. The research reported in the current study was conducted when registration of the Aflasafe product for Nigeria was being pursued (2009–2013). The regulatory agency responsible for registering biopesticides in Nigeria, the National Agency for Food and Drug Administration and Control (NAFDAC), required information on ASA and the potential impact of biocontrol strains on poultry. The objective of the current study was to determine the performance of broilers fed with diets containing maize colonized by either toxigenic or atoxigenic (i.e., an Aflasafe strain) *A. flavus* strains, supplemented with various concentrations of an ASA. Results from this investigation were submitted to NAFDAC to provide evidence that the use of maize from Aflasafe-treated fields had a greater positive effect on broiler performance in comparison to the performance of broilers fed with aflatoxin-contaminated maize supplemented with various concentrations of an ASA. The ASA used in this study was Sim^®^Wall, constituted with beta-glucan, a zeolite, and organic acids. Results strongly indicate that the use of maize treated with atoxigenic strains of Aflasafe has positive effects on the health and productivity of broilers and that ASA does not offer an advantage if aflatoxin-safe crops are available to manufacture feeds.

## 2. Results

There were significant differences in the nutrient composition of diets among and within maize type diets in the starter phase (Table 1). Due to the presence of zeolite, higher (*p* < 0.05) ash content was detected in diets with the highest Sim^®^Wall concentration, regardless of maize type (i.e., control, atoxigenic, or toxigenic maize). Higher contents of both ether extract and crude protein were detected in control maize diets, while the lowest contents occurred in diets prepared with toxigenic maize. There was no trend in which higher values of metabolizable energy were associated with either maize type or Sim^®^Wall. Similar values and trends were detected in diets evaluated at the finisher phase (FP) (Table 2). 

When diets were pooled by maize type, birds fed on diets containing control maize or atoxigenic maize had significantly higher final weight than birds fed with toxigenic maize (Table 3). On the other hand, pooling diets by Sim^®^Wall concentration revealed no differences in final weight (Table 3). Feed intake was not influenced by maize type, but higher weight gain occurred in birds fed with control maize or atoxigenic maize compared to toxigenic maize (Table 3). Higher (*p* < 0.05) FCR occurred in birds fed with toxigenic maize. Mortality in birds fed with toxigenic maize was significantly higher (*p* < 0.05) than the other two maize feed treatments. There were no significant differences in bird mortality among control maize (3.3%) and atoxigenic maize (1.1%) treatments. Overall, the supplementation of Sim^®^Wall (Table 3) in the diets had no effect on final weight, feed intake, weight gain, FCR, or mortality. 

Analysis of diets by maize type revealed significant differences in feed intake and FCR (Table 4). Higher feed intake (*p* < 0.05) occurred by birds fed on control maize with 2000 ppm Sim^®^Wall, atoxigenic maize with 0 ppm Sim^®^Wall, and toxigenic maize with 0 ppm and 2000 ppm Sim^®^Wall (Table 4). FCR was lower (*p* < 0.05) at 0 ppm and 2000 ppm Sim^®^Wall in control maize, at 2000 ppm in atoxigenic maize, and at 1000 ppm in toxigenic maize (Table 4). 

The apparent nutrient digestibility of crude protein, crude fiber, ether extract, dry matter, and ash of excreta of birds fed with control maize or atoxigenic maize was significantly different from those fed on toxigenic maize (Table 5). Sim^®^Wall concentration had no influence (*p* > 0.05) on any nutrient digestibility parameters (Table 5). Within maize types, significant differences were detected only in ash content of birds fed on atoxigenic maize (the highest at 1000 ppm Sim^®^Wall) and in both crude fiber and ether extract of birds fed on toxigenic maize (the highest at 1000 ppm and 2000 ppm Sim^®^Wall; Table 6).

Most of the carcass characteristics and relative organ weight of birds were significantly higher (*p* < 0.05) in birds fed on control maize or atoxigenic maize (Table 7). However, birds fed on toxigenic maize had significantly higher (*p* < 0.05) weight of liver, spleen, and kidney (Table 7). Sim^®^Wall concentration had no influence (*p* > 0.05) on carcass characteristics or organ weight (Table 7). Within and among maize types at different Sim^®^Wall concentrations, there were differences in several parameters (Table 8). Within control maize diets, dressed and breast weights were higher at 0 ppm Sim^®^Wall, while liver and spleen weights were higher at 2000 ppm Sim^®^Wall (Table 8). Within atoxigenic maize diets, breast and drumstick weights were higher at both 0 ppm and 2000 ppm Sim^®^Wall, thigh weight was higher at 0 ppm Sim^®^Wall, and kidney weight was higher at both 1000 ppm and 2000 ppm Sim^®^Wall (Table 8). Within toxigenic maize diets, drumstick weight was higher at 2000 ppm Sim^®^Wall, thigh weight was higher at both 1000 ppm and 2000 ppm Sim^®^Wall, and breast and spleen weights were higher at both 0 ppm and 1000 ppm Sim^®^Wall (Table 8). 

## 3. Discussion

The current study describes the performance of broilers fed with maize treated with two strains of the fungus *A. flavus* and supplemented with different concentrations (0, 1000, or 2000 ppm) of the ASA Sim^®^Wall; maize contained either 0 ppb or 500 ppb aflatoxin B_1_. Nigeria’s biopesticide regulatory agency NAFDAC considered results documented here to register Aflasafe, a biocontrol product for aflatoxin mitigation in maize and groundnut [27]. The active ingredients of Aflasafe are four atoxigenic *A. flavus* strains native to Nigeria. One of the conditions to register the product was to demonstrate both the safety of the product to poultry and its benefit to the poultry industry. Our results strongly indicate that (i) inclusion of maize artificially colonized with atoxigenic *A. flavus* strains (atoxigenic maize) in poultry feeds has positive effects on bird performance in comparison to feeds prepared with maize containing high aflatoxin levels produced by a highly toxigenic *A. flavus* strain (toxigenic maize), and that (ii) poultry performance is similar when fed with either atoxigenic maize or aflatoxin-safe untreated maize (control maize). Almost all evaluated parameters of birds fed with atoxigenic maize or control maize were consistently similar. Also, this is the first report that compares the use of maize treated with an atoxigenic *A. flavus* strain to using an ASA to reduce broiler exposure to aflatoxins. We found that, even when supplementing the ASA at the highest concentration, performance of birds fed on toxigenic maize was inferior to that of birds fed on atoxigenic maize or control maize. 

Poultry feeds manufactured in Nigeria contain a high proportion of maize, and its aflatoxin content is typically high [12,13]. High aflatoxin concentrations in feeds, as those found in Nigeria, have several adverse effects on birds, ranging from low productivity to increased mortality [7]. Approximately two million metric tons of maize were used to manufacture feeds in Nigeria during 2013/2014. With Nigeria’s projected population growth, the requirement of maize for feed is expected to increase considerably as a result of higher demand for poultry products [11]. Since maize produced in Nigeria is at risk of aflatoxin contamination [34,35,36,37], coupled with the fact that most farmers and processors typically do not implement aflatoxin management strategies, poultry productivity in Nigeria is highly likely hindered by aflatoxins. If poultry industries had access to feeds manufactured with aflatoxin-safe crops, poultry performance would improve, leading to higher profits. 

Aflatoxin in feed is an important determinant of poultry performance [5]. Overall, birds fed on either atoxigenic maize or control maize had higher weight, lower FCR, and lower mortality than birds fed on toxigenic maize (Table 3 and Table 4). This was reflected by the poor quality of excreta of birds fed on toxigenic maize (Table 5 and Table 6). As expected, birds fed on toxigenic maize had lower weight of most organs and increased weight of liver and kidney (Table 7 and Table 8). Monson et al. reviewed the effects of aflatoxins in tissues of different avian species, including *Gallus gallus domesticus* [7]. Such negative effects include formation of DNA and RNA adducts inducing DNA mutations, hepatoxicity, immunotoxicity, and intestinal toxicity, among others. These effects are assumed to have affected the birds evaluated in the current study. 

The ASA used in this study would be of little or no use to improve overall poultry performance (Table 3, Table 4, Table 7 and Table 8). The weight of the evaluated organs was not influenced by Sim^®^Wall concentration (Table 7). In some cases, the relative weight of certain organs was higher either in the absence of ASA or at 1000 ppm, while other organs had higher weight either at 1000 ppm and/or 2000 ppm. On the other hand, higher organ weight was detected in the absence of ASA in both atoxigenic maize and control maize. Clearly, using aflatoxin-safe maize had more significant positive impacts on poultry performance than using the evaluated ASA. As discussed in two recent review articles [16,38], using aflatoxin-safe crops is a better management option than using an ASA because of their limitations such as higher FCR, higher feed rations, accumulation of antibiotics in tissues, and interactions with nutrients, among others. Several of those limitations were found in the current study. If poultry producers had no access to aflatoxin-safe maize and the evaluated ASA was the sole option to reduce poultry aflatoxin exposure, the benefits would have been marginal or absent. However, more effective but costlier ASAs are also available in the market.

Incorporation of atoxigenic maize, which was previously treated with one of the atoxigenic *A. flavus* strains composing the biocontrol product, into diets had no negative effects on growth performance of birds in comparison to birds fed on diets containing control maize. The maize used to prepare the control maize diets did not contain aflatoxins but was associated with aflatoxin-producing fungi (data not shown). Maize produced in the tropics and subtropics is virtually always associated with aflatoxin-producing fungi because the genus *Aspergillus* is ubiquitous in those environments [39]. The aflatoxin content in maize is dictated by several factors that include poor pre- and post-harvest management practices, high prevalence of highly toxigenic aflatoxin-producing fungi, and conducive environmental conditions, among others [40]. The maize grain used in the current study was produced under appropriate, optimal agricultural practices in the IITA-Ibadan research farm. Those conditions are typically not found across Nigeria, and a reflection of this is that feeds in this nation are associated with aflatoxin-producing fungi at high densities and frequently contain high aflatoxin concentrations [12,13].

Treatment with the biological control product Aflasafe in the field to produce aflatoxin-safe maize grains subsequently used for manufacturing feeds offers a practical solution to the burden caused by aflatoxins to poultry producers. According to recent reviews [16,38], it seems that there is a long path before ASA consistently and effectively counteracts the toxic effects of aflatoxins to livestock, including poultry. In Nigeria, many poultry producers are procuring maize treated with Aflasafe because, after testing the aflatoxin-safe maize in their farms, they realized that it provides greater benefits than using non-treated maize or any of the ASAs available in the market [27,28]. Certain classes of feed manufacturers and poultry producers were willing to pay average premiums of 4.9% to 30.9% for maize with <10 ppb aflatoxin content, and even larger premium for maize with <4 ppb aflatoxin content, relative to maize with <20 ppb aflatoxin content [41].

## 4. Conclusions

Findings from the current study were part of a dossier submitted to NAFDAC for registration of the biocontrol product Aflasafe. These findings demonstrated that biocontrol contributes to the public good because the atoxigenic *A. flavus* strains composing the product are not harmful to poultry, and using crops treated with Aflasafe as major components of poultry feeds substantially improves poultry health and productivity in comparison to using an ASA. Biocontrol is being used on a large scale across Nigeria by tens of thousands of farmers (www.agresults.org). A large portion of the maize produced in treated fields and tested for aflatoxin standard compliance is processed into feeds used by poultry producers [27,28]. It is expected that the maize area treated with biocontrol will enhance considerably due to increased private sector participation in the manufacturing and distribution of Aflasafe. This will result in more poultry businesses having access to aflatoxin-compliant crops and higher profits as a result of improved poultry performance. Finally, consumers will have access to safe and nutritious poultry products as a result of the increased availability of aflatoxin-safe crops.

## 5. Materials and Methods

### 5.1. Broiler Management 

A total of 270 unsexed one-day-old broilers (*G. gallus domesticus*; Marshall strain) were purchased from a hatchery in Abeokuta, Ogun State, Nigeria. Each broiler was randomly assigned to one of 27 pens on a deep litter system at the Poultry Unit of the Teaching and Research Farm of the Federal University of Agriculture, Abeokuta. Ten broilers were assigned to each pen, which had a floor area of 2.4 m^2^. Prior to assignment of broilers to pens, pens were thoroughly washed and disinfected with 1% Morigad Lysol (Morison Industries, Lagos, Nigeria), a broad-spectrum disinfectant for veterinary use, and they were left to dry for three days. Pens were then fumigated with 40% formalin amended with potassium permanganate crystals. For brooding, heat sources were provided from both charcoal and electric bulbs. Wood shavings were spread on floors of the pens for bedding and insulation. Routine vaccination (New Castle disease and Gumboro vaccines, National Veterinary Research Institute, VOM, Plateau State, Nigeria), medication (Coccidiostat; Totralvet 2.5%), and vitamins (Lytavite Plus Dextrose) were administered at prophylactic levels as described by Oluyemi and Roberts [42].

### 5.2. Maize Used to Prepare Diets

Grains from the mid-maturing maize variety ACCR-9931-SR-Y, grown by IITA-Ibadan Maize Breeding Unit, were used as the maize component of all evaluated diets. Aflatoxin content of maize grains was determined by extracting aflatoxins and using thin-layer chromatography as previously described [32], and no aflatoxins were detected (limit of detection (LOD) = 0.1 ppb total aflatoxins). 

Three types of maize were used in the study: (1) non-treated maize, (2) maize colonized by atoxigenic *A. flavus* strain La3279, and (3) maize colonized by toxigenic *A. flavus* strain La3228; in this study, the maize types are referred to as control maize, atoxigenic maize, and toxigenic maize, respectively (Table 1 and Table 2). To prepare 350 kg of atoxigenic maize, 2-kg maize batches were surface-sterilized by immersion in hot water (40 s, 80 °C), blotted free of moisture in a safety cabinet, and inoculated with the atoxigenic strain. The same procedure was used to prepare 350 kg of toxigenic maize, but inoculation was conducted with the toxigenic strain. All inoculated grains were incubated for seven days (38 °C). Spores from colonized grains were washed off, and grains were dried to circa 8% moisture content at 48 °C in an oven. For both sets of grains, aflatoxin content was quantified as above. Aflatoxins were not detected in the atoxigenic maize while, on an average, 947 ppb aflatoxin B_1_ and 74 ppb aflatoxin B_2_ were detected in toxigenic maize. Both sets of maize were independently blended with a laboratory mill (Waring Commercial, Torrington, CT, US) for incorporation in the different diets (see below). Toxigenic maize was combined with aflatoxin-free maize to reach 500 ppb aflatoxin B_1_. 

### 5.3. Aflatoxin-Sequestering Agent

The ASA used was Sim^®^Wall, a feed additive containing beta-glucan of yeast origin, purified and activated clinoptilolite (a zeolite), and organic acids (formic and acetic acids, and propionate). Sim^®^Wall (Simbiyotek Biological Products International, Istanbul, Turkey) was incorporated into the tested diets at one of the following concentrations: 0, 1000, or 2000 ppm. 

### 5.4. Tested Diets

Nine diets were prepared for evaluation at the starter phase (SP; week 1–4) using one of the three maize types amended with various concentrations of Sim^®^Wall in a 3 × 3 factorial experimental design. Diets 1, 2, and 3 contained control maize supplemented with 0, 1000, and 2000 ppm Sim^®^Wall, respectively. Diets 4, 5, and 6 contained atoxigenic maize with 0, 1000, and 2000 ppm Sim^®^Wall, respectively. Diets 7, 8, and 9 contained toxigenic maize supplemented with 0, 1000, and 2000 ppm Sim^®^Wall, respectively. 

Per kilogram, all nine diets evaluated at SP contained 500 g of the corresponding maize type, wheat offal (112 g), soybean meal (340 g), bone meal (30 g), oyster shell (10 g), vitamin (Vit)/mineral premix (2 g (Vit A: 12,000 IU, Vit D_3_: 2,500 IU, Vit E: 30 mg, Vit K_3_: 2 mg, Vit B_1_: 2.25 mg, Vit B_2_: 6 mg, Vit B_6_: 4.5 mg, Vit B_12_: 0.015 mcg, niacin: 40 mg, pantothenic acid: 15 mg, folic acid: 1.5 mg, biotin: 0.05 mcg, manganese: 80 mg, iron: 20 mg, zinc: 50 mg, copper: 5 mg, cobalt: 0.5 mg, iodine: 1 mg, selenium: 0.2 mg, choline chloride: 300 mg, and antioxidants: 125 mg)), salt (2.5 g), methionine (1 g), and lysine (2 g).

For the finisher phase (FP; week 5–8), nine diets were prepared with maize type and Sim^®^Wall concentrations as above. Per kilogram, diets contained 540 g of the corresponding maize type, wheat offal (152 g), soybean meal (260 g), bone meal (30 g), oyster shell (10 g), vitamin/mineral premix (2 g; see above), salt (2.5 g), methionine (1 g), and lysine (2 g).

In both SP and FP, there were three replicates per tested diet, and each replicate was composed of 10 broilers. The birds were maintained on the assigned diet throughout the experiment. All diets were formulated to meet the nutritional requirements stated by the National Research Council of the US [8]. Neither antibiotics nor growth promoters were administered to broilers. The birds were continuously exposed to light during the three-week brooding period. Feed and water were provided ad libitum throughout the eight-week rearing period. 

### 5.5. Data Collection

Birds were weighed individually on a weekly basis. Feed intake was measured weekly. Those values were used to calculate the FCR and weight gain/feed intake (Table 3 and Table 4). Mortality was recorded throughout the experiment (Table 3 and Table 4). 

At the end of the FP, six broilers per diet (two per replicate) were randomly selected and placed in clean, disinfected, and specially designed metabolic steel cages for six days to conduct nutrient digestibility trials. Broilers were given three days of acclimatization and were fed with a regular supply (about 140 g) of their assigned diet. For example, broilers on diet 1 were also fed on diet 1 while in the metabolic cage. Excreta samples from each bird were collected daily at 5:00 p.m., weighed, and dried in a drying cabinet (LEEC Drying Cabinet, model FCX1, Nottingham, UK) at 60 °C for 72 h. Caution was taken to ensure that the excreta were not contaminated with feathers. The cumulative samples were pooled together and finely ground for laboratory analysis. The feed and excreta samples were analyzed for proximate composition, dry matter, metabolizable energy, crude protein, crude fiber, ether extract, and ash according to the Official Methods of Analysis recommended by the Association of Official Analytical Chemists [43]. The values obtained were used to calculate the percentage apparent digestibility of nutrients. 

For carcass analyses at the end of FP, a bird per replicate was randomly selected and sacrificed by cervical dislocation, plucked, and eviscerated. The live weight, plucked weight, eviscerated weight, dressed weight, weight of cut parts, and offal weight were measured using a sensitive scale balance (A&D GF6000 series, San Jose, CA, US). Weights of cut parts and offal were expressed as percentage of live weight of birds.

### 5.6. Safety Measures

The farm personnel adhered to strict safety measures that included wearing a 3M™ Particulate Respirator 8210, N95 (3M, Saint Paul, MN, US), latex or leather gloves, safety apparel, and boots during both preparation of diets and daily routine management of birds. The Poultry Unit Management of the Teaching and Research Farm of the Federal University of Agriculture gave ethical approval to carry out the study. Birds fed on toxigenic diets were sacrificed and buried at the expiration of the trial as required by the Poultry Management Unit.

### 5.7. Statistical Analyses

Data collected were analyzed by analysis of covariance (ANCOVA) and by analysis of variance (ANOVA) using SAS statistical package (version 9.4, SAS Institute Inc, Cary, NC, USA). ANCOVA was performed for treatment influence on performance and carcass characteristics of broilers, to account for variation in the initial starting weight of the birds. ANOVA was performed for data from nutrient composition and digestibility studies. Treatment means were separated using the Student–Newman–Keuls test (*α* = 0.05).

## Figures and Tables

**Table 1:** Nutrient composition of diets fed to broilers at starter phase ^A^.

Parameter	Control Maize			Atoxigenic Maize			Toxigenic Maize		
	Diet 1	Diet 2	Diet 3	Diet 4	Diet 5	Diet 6	Diet 7	Diet 8	Diet 9
Sim^®^Wall (ppm)	0	1000	2000	0	1000	2000	0	1000	2000
*Determined analysis*									
Ash (g/kg)	93.8 b	94.5 b	98.3 a	92.1 c	93.6 b	97.3 a	82.5 e	88.0 d	97.3 a
Crude fiber (g/kg)	38.1 a	38.3 a	37.5 b	36.9 c	37.4 b	37.4 b	35.0 d	37.5 b	37.4 b
Ether extract (g/kg)	35.9 b	35.8 c	36.5 a	35.0 e	34.5 f	35.5 d	33.0 i	33.9 g	33.3 h
Crude protein (g/kg)	22.3 b	23.0 a	22.0 c	21.2 d	20.4 f	20.8 e	17.9 i	19.7 h	19.8 g
ME ^B^ (MJ/kg)	10.8 f	11.6 b	11.7 a	11.3 d	11.4 c	11.4 c	11.7 a	11.1 e	11.6 b

^A^ Means with the same lowercase letter (i.e., a to i) in the same row are not significantly different based on a Student–Newman–Keuls test (*α* = 0.05). Control maize: untreated maize with no detectable aflatoxin levels; atoxigenic maize: maize treated with an atoxigenic *Aspergillus flavus* strain of the biocontrol product Aflasafe; toxigenic maize: maize treated with an aflatoxin-producing *A. flavus* strain, containing 500 ppb aflatoxin B_1_. ^B^ ME: metabolizable energy.

**Table 2:** Nutrient composition of diets fed to broilers at finisher phase ^A^.

Parameter	Control Maize			Atoxigenic Maize			Toxigenic Maize		
	Diet 1	Diet 2	Diet 3	Diet 4	Diet 5	Diet 6	Diet 7	Diet 8	Diet 9
Sim^®^Wall (ppm)	0	1000	2000	0	1000	2000	0	1000	2000
*Determined analysis*									
Ash (g/kg)	9.3 de	9.0 f	10.2 a	8.6 g	9.2 e	9.5 c	8.8 g	9.4 cd	9.8 b
Crude fiber (g/kg)	4.8 b	4.9 a	4.9 a	4.7 c	4.7 c	4.7 c	4.3 f	4.5 d	4.4 e
Ether extract (g/kg)	3.7 a	3.5 c	3.6 b	3.6 b	3.6 b	3.5 c	3.4 d	3.4 d	3.4 d
Crude protein (g/kg)	21.4 a	20.8 e	21.2 c	20.9 d	20.8 e	21.3 b	17.9 h	18.4 g	18.8 f
ME ^B^ (MJ/kg)	11.9 d	12.1 c	12.3 b	11.6 c	11.6 e	11.9 d	10.9 g	11.1 f	12.9 a

^A^ Means with the same lowercase letter (i.e., a to h) in the same row are not significantly different based on a Student–Newman–Keuls test (α = 0.05). Control maize: untreated maize with no detectable aflatoxin levels; atoxigenic maize: maize treated with an atoxigenic *Aspergillus flavus* strain of the biocontrol product Aflasafe; toxigenic maize: maize treated with an aflatoxin-producing *A. flavus* strain, containing 500 ppb aflatoxin B_1_. ^B^ ME: metabolizable energy.

**Table 3:** Performance of broiler chickens fed with feed containing various maize types and concentrations of the aflatoxin-sequestering agent Sim^®^Wall ^A^.

Parameters	Maize type			Sim^®^Wall		
	Control	Atoxigenic	Toxigenic	0 ppm	1000 ppm	2000 ppm
Initial weight (g)	39.2 a	38.9 a	37.6 b	38.7 ab	37.9 b	39.2 a
Final weight (g)	1785 a	1831 a	1118 b	1577 a	1512 a	1645 a
Feed intake (g)	142.7 a	139.6 a	147.0 a	146.1 a	138.7 a	144.5 a
Daily weight gain (g)	43.0 a	43.7 a	26.7 b	37.2 a	35.9 a	40.2 a
FCR ^B^	3.3 b	3.1 b	5.4 a	4.0 a	4.1 a	3.8 a
Mortality (%)	3.3 b	1.1 b	11.2 a	5.7 a	3.3 a	6.7 a

^A^ Means with the same lowercase letter (i.e., a to b) in the same row are not significantly different based on a Student–Newman–Keuls test (*α* = 0.05). Analyses were done separately by maize type and Sim^®^Wall. Control maize: untreated maize with no detectable aflatoxin levels; atoxigenic maize: maize treated with an atoxigenic *Aspergillus flavus* strain of the biocontrol product Aflasafe; toxigenic maize: maize treated with an aflatoxin-producing *A. flavus* strain, containing 500 ppb aflatoxin B_1_. ^B^ FCR: feed conversion ratio.

**Table 4:** Performance of broiler chickens fed with three maize types, each containing various concentrations of an aflatoxin-sequestering agent ^A, B^.

Parameter	Control Maize			Atoxigenic Maize			Toxigenic Maize		
	Diet 1	Diet 2	Diet 3	Diet 4	Diet 5	Diet 6	Diet 7	Diet 8	Diet 9
Final weight (g)	1783 a	1683 a	1890 a	1829 a	1765 a	1900 a	1119 b	1088 b	1145 b
Feed intake (g)	94.2 c∫	96.5 c∫	100.2 b*	96.5 c*	93.9 c#	94.2 c∫	100.7 b*	94.1 c∫	103.6 a*
Daily weight gain (g)	42.2 a	39.9 a	46.7 a	43.3 a	41.8 a	45.9 a	26.1 b	25.8 b	28.0 b
FCR ^C^	2.9 d∫	3.2 c*	2.9 d∫	2.9 d∫	2.9 d*	2.7 e#	5.0 a*	4.8 b∫	5.1 a*
Mortality (%)	0.0 a	3.3 a	6.7 a	3.3 a	0.0 a	0.0 a	13.7 a	6.7 a	13.3 a

^A^ Means with the same lowercase letter (i.e., a to e) in the same row are not significantly different based on a Student–Newman–Keuls test (*α* = 0.05). Means within respective maize types with different symbols (*, ∫, #) differ statistically (* > ∫ > #) from one another (*α* = 0.05); absence of those symbols indicates that diets within maize types are not significantly different. Control maize: untreated maize with no detectable aflatoxin levels; atoxigenic maize: maize treated with an atoxigenic *Aspergillus flavus* strain of the biocontrol product Aflasafe; toxigenic maize: maize treated with an aflatoxin-producing *A. flavus* strain, containing 500 ppb aflatoxin B_1_. ^B^ Diets 1, 4, 7 = no Sim^®^Wall; diets 2, 5, 8 = with 1000 ppm Sim^®^Wall; diets 3, 6, 9 = with 2000 ppm Sim^®^Wall. ^C^ FCR: feed conversion ratio.

**Table 5:** Apparent nutrient digestibility observed in excreta of birds fed with different maize types and at different aflatoxin-sequestering agent concentrations examined separately ^A^.

Parameters (%)	Maize type			Sim^®^Wall		
	Control	Atoxigenic	Toxigenic	0 ppm	1000 ppm	2000 ppm
Dry matter	78.8 a	79.3 a	60.4 b	72.4 a	73.0 a	73.1 a
Crude protein	70.8 a	70.6 a	53.8 b	65.1 a	64.7 a	65.4 a
Crude fiber	67.3 a	66.4 a	56.6 b	62.8 a	63.8 a	63.8 a
Ether extract	70.9 a	70.5 a	58.6 b	65.9 a	66.7 a	67.3 a
Ash	63.1 a	62.1 a	54.8 b	58.4 a	60.5 a	61.1 a

^A^ Means with the same lowercase letter in the same row (i.e., a to b) are not significantly different based on a Student–Newman–Keuls test (*α* = 0.05). Analyses were done separately by maize type and Sim^®^Wall. Control maize: untreated maize with no detectable aflatoxin levels; atoxigenic maize: maize treated with an atoxigenic *Aspergillus flavus* strain of the biocontrol product Aflasafe; toxigenic maize: maize treated with an aflatoxin-producing *A. flavus* strain, containing 500 ppb aflatoxin B_1_.

**Table 6:** Apparent nutrient digestibility observed in birds fed with feed containing various maize types, each containing different concentrations of an aflatoxin-sequestering agent ^A, B^.

Parameter (%)	Control Maize			Atoxigenic Maize			Toxigenic Maize		
	Diet 1	Diet 2	Diet 3	Diet 4	Diet 5	Diet 6	Diet 7	Diet 8	Diet 9
Ash	62.61 a	61.31 a	65.50 a	60.81 a∫	64.09 a*	61.43 a∫	51.73 c	56.05 b	56.51 b
Crude fiber	66.94 a	67.43 a	67.60 a	66.67 a	66.41 a	66.01 a	54.71 c∫	57.52 b*	57.64 b*
Ether extract	71.77 a	70.59 a	70.26 a	70.65 a	70.11 a	70.66 a	55.43 c∫	59.31 b*∫	60.97 b*
Crude protein	70.73 a	71.18 a	70.63 a	70.73 a	70.31 a	70.72 a	53.74 b	52.68 b	54.83 b
Dry matter	78.28 a	78.48 a	79.60 a	78.62 a	79.58 a	79.58 a	60.21 b	61.07 b	59.99 b

^A^ Means with the same lowercase letter in the same row (i.e., a to c) are not significantly different based on a Student–Newman–Keuls test (*α* = 0.05). Means within respective maize types with different symbols (*, ∫) differ statistically (* > ∫) from one another (*α* = 0.05); absence of those symbols indicates that diets within maize types are not significantly different. Control maize: untreated maize with no detectable aflatoxin levels; atoxigenic maize: maize treated with an atoxigenic *Aspergillus flavus* strain of the biocontrol product Aflasafe; toxigenic maize: maize treated with an aflatoxin-producing *A. flavus* strain, containing 500 ppb aflatoxin B_1_. ^B^ Diets 1, 4, 7 = no Sim^®^Wall; diets 2, 5, 8 = with 1000 ppm Sim^®^Wall; diets 3, 6, 9 = with 2000 ppm Sim^®^Wall.

**Table 7:** Carcass characteristics and relative organ weight of broiler chickens fed with feed containing various maize types and concentrations of an aflatoxin-sequestering agent ^A^.

Parameters (%)	Maize type			Sim^®^Wall		
	Control	Atoxigenic	Toxigenic	0 ppm	1000 ppm	2000 ppm
Dressed weight	68.79 a	70.16 a	58.34 b	66.36 a	65.68 a	65.23 a
Breast	20.02 a	21.09 a	14.58 b	19.58 a	18.61 a	17.52 a
Drumstick	9.37 a	10.26 a	8.31 b	8.88 a	9.51 a	9.54 a
Thigh	9.85 a	10.12 a	8.28 b	9.01 a	9.71 a	9.54 a
Abdominal fat	0.83 a	0.94 a	0.00 b	0.62 a	0.65 a	0.51 a
Liver	1.76 b	1.76 b	3.39 a	2.28 a	2.38 a	2.24 a
Spleen	0.16 b	0.14 b	0.21 a	0.16 a	0.17 a	0.17 a
Kidney	0.53 b	0.61 b	0.81 a	0.63 a	0.66 a	0.66 a

^A^ Means with the same lowercase letter in the same row (i.e., a to b) are not significantly different based on a Student–Newman–Keuls test (*α* = 0.05). Analyses were done separately by maize type and Sim^®^Wall. Control maize: untreated maize with no detectable aflatoxin levels; atoxigenic maize: maize treated with an atoxigenic *Aspergillus flavus* strain of the biocontrol product Aflasafe; toxigenic maize: maize treated with an aflatoxin-producing *A. flavus* strain, containing 500 ppb aflatoxin B_1_.

**Table 8:** Characteristics and relative organ weight of broiler chickens fed with feed containing various maize types, each containing different concentrations of an aflatoxin-sequestering agent ^A, B^.

Parameter (%)	Control Maize			Atoxigenic Maize			Toxigenic Maize		
	Diet 1	Diet 2	Diet 3	Diet 4	Diet 5	Diet 6	Diet 7	Diet 8	Diet 9
Dressed weight	69.66 ab*	69.21 ab∫	67.50 b#	69.93 ab	68.25 b	72.29 a	59.50 c	59.59 c	55.92 d
Breast	22.15 a*	19.89 b∫	18.04 c#	21.05 ab*∫	20.49 ab∫	21.74 a*	15.53 d*	15.45 d*	12.77 e∫
Drumstick	9.88 ab	9.67 abc	8.55 c	10.41 a*	9.93 ab∫	10.45 a*	6.37 d#	8.93 bc∫	9.62 abc*
Thigh	10.56 a	10.53 a	8.47 a	10.00 a*	9.78 a∫	10.60 a∫	6.46 b∫	8.81 a*	9.56 a*
Abdominal fat	1.19 a	0.79 abc	0.50 c	0.66 bc	1.16 a	1.02 ab	0.00 d	0.00 d	0.00 d
Liver	1.50 b#	1.81 b∫	1.96 b*	1.68 b	1.86 b	1.73 b	3.65 a	3.47 a	3.04 a
Spleen	0.13 b∫	0.14 b∫	0.20 a*	0.14 b	0.13 b	0.13 b	0.22 a*	0.23 a*	0.17 b∫
Kidney	0.57 a	0.53 a	0.49 a	0.47 a∫	0.73 a*	0.64 a*	0.85 a	0.71 a	0.86 a

^A^ Means with the same lowercase letter (e.g., a to e) in the same row are not significantly different based on a Student–Newman–Keuls test (*α* = 0.05). Means within respective maize types with different symbols (*, ∫, #) differ statistically (* > ∫ > #) from one another (*α* = 0.05); absence of those symbols indicates that diets within maize types are not significantly different. Control maize: untreated maize with no detectable aflatoxin levels; atoxigenic maize: maize treated with an atoxigenic *Aspergillus flavus* strain of the biocontrol product Aflasafe; toxigenic maize: maize treated with an aflatoxin-producing *A. flavus* strain, containing 500 ppb aflatoxin B_1_. ^B^ Diets 1, 4, 7 = no Sim^®^Wall; diets 2, 5, 8 = with 1000 ppm Sim^®^Wall; diets 3, 6, 9 = with 2000 ppm Sim^®^Wall.
